# Ceria nanoparticles deposited on graphene nanosheets for adsorption of copper(II) and lead(II) ions and of anionic species of arsenic and selenium

**DOI:** 10.1007/s00604-018-2806-6

**Published:** 2018-04-23

**Authors:** Anna Baranik, Anna Gagor, Ignasi Queralt, Eva Marguí, Rafal Sitko, Beata Zawisza

**Affiliations:** 1 Institute of Chemistry, University of Silesia, Szkolna 9, 40-006 Katowice, Poland; 2 Institute of Low Temperature and Structure Research, Polish Academy of Sciences, P.O. Box 1410, 50-950 Wrocław, Poland; 3 Department of Geosciences, Institute of Environmental Assessment and Water Research, IDAEA-CSIC, Jordi Girona St., 18-26, 08034 Barcelona, Spain; 4 Department of Chemistry, University of Girona, Faculty of Sciences, C/M.Aurèlia Campmany, 69, Girona, Spain

**Keywords:** Nanocomposite, Graphene, Trace analysis, Sorption, Speciation, EDXRF, DSPME, DMSPE

## Abstract

A nanocomposite prepared from graphene nanosheets and cerium nanoparticles (G/CeO_2_) was applied to the extraction of Se(IV), As(V), As(III), Cu(II) and Pb(II). The structure of G/CeO_2_ was investigated by scanning electron microscopy, X-ray diffraction and Raman spectroscopy. The optimal pH values for extraction are 4.0 for As(V), 3.0 for Se(IV), and 6.0 for both Cu(II) and Pb(II). The maximum adsorption capacity of G/CeO_2_ (expressed as mg·g^−1^) were calculated by the Langmuir model and are found to be 8.4 for As(V), 14.1 for Se(IV), 50.0 for Cu(II) and 75.6 for Pb(II). The sorbent was applied to dispersive solid phase microextraction prior to direct quantitation by energy-dispersive X-ray fluorescence spectrometry without the need for prior elution. The limits of detection (in ng·mL^−1^ units) are 0.10 for As(V), 0.11 for Se(IV), 0.19 for Cu(II) and 0.21 for Pb(II). The precisions (RSDs) are <4.5%. The accuracy of the method (1 - 4%) was verified by analysis of the certified reference material (CRM 1640a - natural water). The method was successfully applied in ultratrace element determination and to the speciation of selenium in environmental waters.

**Electronic supplementary material:**

The online version of this article (10.1007/s00604-018-2806-6) contains supplementary material, which is available to authorized users.

**Graphical abstract:**
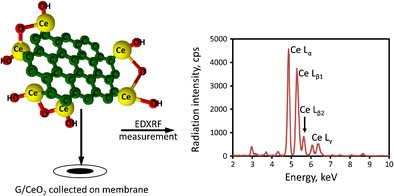
The method gives possibility of simultaneous preconcentration and determination in environmental waters of both anionic (As(V) and Se(IV)) and cationic (Cu(II) and Pb(II)) forms of selected metals using graphene nanosheets and cerium nanoparticles. Se(IV) can be selective determined in the presence of Se(VI).

## Introduction

Solid phase extraction (SPE) is one of the most commonly used procedure in analyte preconcentration and for removing impurities from samples. The possibility to use a broad range of sorbent materials, chelating reagents and eluents make this method very attractive for sample treatment. Moreover, SPE procedures can be easily implemented and controlled in flow systems to perform appropriate sample pretreatment. The mechanism of sorption depends on the nature of a given sorbent and may include simple adsorption, complexation or ion-exchange. The choice of solid material for enrichment should be based on the content of the analyte, sample matrix and technique used for final detection. Graphene (G) and graphene oxide (GO) have several advantages as adsorbents but their practical use in classical SPE can be hampered because their reduced particle sizes. Nanoparticles can cause high pressure in the SPE column as well as they can escape from the SPE column, particularly under high pressure [1]. Solid phase microextraction **(**SPME) is currently one of the most popular green techniques used for sample preparation in analytical chemistry [2]. A wide choice of nanosorbents allows them to be used in organic and inorganic analyses [3]. Moreover, the surface functionality of these sorbents can be easily modified to achieve selective sample extraction [4,5]. An alternative to ordinary SPE is magnetic SPE (MSPE) [6,7]. In MSPE, analytes are preconcentrated from aqueous solutions using magnetic adsorbents. Magnetic adsorbents are dispersed in an aqueous solution containing analytes. The analytes adsorbed on the magnetic adsorbent are then collected by a magnetic field. Fe_3_O_4_ particles are widely used as magnetic adsorbent in SPE, but they suffer from several limitations such as the aggregation, oxidization, and instability at pH < 4, which significantly reduce their superparamagnetism. To cope with such problems an interesting approach is the use of dispersive solid phase microextraction (DSPME). DSPME is a notably efficient extraction technique. The most relevant advantages of this procedure over the traditional SPE are a higher extraction efficiency, short extraction time, reduced solvent consumption and simplified extraction process. In DSPME the dispersion and extraction process are assisted by an external energy such as e.g. sonication, and the extraction is carried out in the bulk solution with no need for a cartridge, disk or column. Bonding of the analytes from a solution onto a suitable sorbent is mainly performed via adsorption. The ideal sorbents in DSPME procedures are nanomaterials due to their large adsorption area and their high equilibrium speed. Nevertheless, most of published methods using nanomaterials as sorbents are dedicated to the asorption/determination of positive ions like Cr(III), Fe(III), Co(II), Ni(II), Cu(II), Zn(II), Cd(II) and Pb(II) [8–10] and organic compounds [3,11]. Sorption and determination of anionic forms of elements is much more difficult, but it is also necessary and important. The ultrahigh specific surface area of graphene is responsible for its high chemical activity as well as high adsorption capacity. However, metal ions can be often adsorbed on graphene nanosheets only as hydrophobic complexes using chelating agents. Therefore, the development of functionalized graphene is recommended particularly in the context of the anionic species adsorption as well as the enhancement of its selectivity.

In this study, a nanocomposite of graphene nanosheets aggregated cerium nanoparticles (G/CeO_2_) was examined as a new adsorbent for the extraction of arsenic(V), selenium(IV), copper(II) and lead(II) without the using of chelating agent. The nano-sizes of ceria nanoparticles were attached successfully on the graphene nanosheets by using a commercially non-ionic surfactant agent (Triton-X100). Moreover a new method based on the combination of DSPME and EDXRF was developed for the determination of both anionic as well as cationic species of selected elements at a ultratrace level.

## Experimental

### Reagents and solutions

As(V), Se(IV), Cu(II) and Pb(II) stock solutions of 1 mg∙mL^−1^ were purchased from Merck (Darmstadt, Germany, www.merckmilipore.com). Humic acid was purchased from Sigma-Aldrich (Steinheim, Germany, www.sigmaaldrich.com); graphene nanopowder 8 nm (purity 99.99% and the flakes size 8 nm) was acquired from graphene supermarket (New York, The United State, www.graphene-supermarket.com); nitric acid (65%, Suprapur), chloric acid (35-38% p.a.), ammonium hydroxide solution (25%, p.a.), potassium permanganate (p.a.), cerium (III) nitrate hexahydrate (p.a.), Triton-X-100, sodium hydroxide (p.a.), lead(II) nitrate (p.a.), copper(II) nitrate trihydrate (p.a.), sodium nitrate (p.a.), potassium nitrate (p.a.), calcium nitrate tetrahydrate (p.a.), magnesium nitrate hexahydrate (p.a.), iron (III) nitrate nanohydrate (p.a.), aluminium nitrate nanohydrate (p.a.), buffer solution (pH 4.00 and pH 7.00) were purchased from Avantor Performance Materials Poland S.A. (Gliwice, Poland, www.poch.com.pl). Standard solutions were diluted with high purity water obtained from Milli-Q system (Millipore, Molsheim, France, www.merckmilipore.com). Filters (pore size 0.45 μm) were purchased from Merck (Darmstadt, Germany, www.merckmilipore.com). The Certified Reference Material (natural water 1640a) was acquired from the National Institute of Standards and Technology USA (Gaithersburg, The United State, www.nist.gov).

### Instruments

SEM micrographs as well as sample composition information were obtained using a FEI Nova NanoSEM 230 microscope (Oregon, The United State,www.fei.com). EDS spectra were acquired and analysed using an EDAX Pegasus XM4 spectrometer with SDD Apollo 40 detector (New Jersey, The United State, the www.edax.com).

Powder diffraction data (XRD) (PANalytical, Almelo, The Netherlands, www.panalytical.com/Home.htm) were collected on X’Pert PRO X-ray diffractometer with PIXcel ultrafast line detector and Soller slits for Cu Kα radiation. The measurements were done in Bragg-Brentano geometry.

The Raman spectra (Renishaw, New Mills, Wotton - under - Edge Gloucestershire, United Kingdom, http://www.renishaw.com/en/1030.aspx) were measured at room temperature using a RenishawInVia Raman spectrometer equipped with a confocal DM 2500 Leica optical microscope, a thermoelectrically cooled Ren Cam CCD detector and a diode laser operating at 830 nm.

Inductively coupled plasma atomic emission spectroscopy (ICP-OES) (Spectro Analytical Instruments GmbH, Kleve, Germany, www.spectro.com) measurements were performed using a SpectroBlue FMS16 spectrometer with inductively coupled plasma (ICP) excitation (Spectro Analytical Instruments). A charge coupled device detector is installed in this spectrometer. The following operation parameters were used: plasma power – 1.45 kW; coolant gas – Ar, 12 L∙min^−1^; auxiliary gas – Ar, 1 L∙min^−1^; nebulizer gas – Ar, 1 L∙min^−1^; nebulizer pressure – 3.2 bar; nebulizer-cross-flow type; sample uptake rate – 2 mL∙min^−1^; and wavelength – 193.759 nm, 196.090 nm, 324.754 nm and 220.353 nm for As, Se, Cu and Pb, respectively.

Energy-dispersive X-ray fluorescence spectrometry (EDXRF) measurements were performed using Epsilon 3 spectrometer (PANalytical, Almelo, The Netherlands, www.panalytical.com) with a Rh target X-ray tube (50 μm Be window and max. Power of 9) and thermoelectrically cooled silicon drift detector (SDD) with 8 μm Be window and resolution of 135 eV at 5.9 keV. The spectrometer is equipped with spinner and five primary filters that can be selected to improve measuring conditions for determined elements.

### Synthesis of G/CeO_2_

1 g of graphene and 300 mg of the non-ionic surfactant Triton-X-100 (surfactant was added for dispersing hydrophobic graphene in aqueous solutions) were dispersed in 20 mL of water for 1 h. Then, 20 mL of cerium(III) nitrate hexahydrate (0.035 g∙mL^−1^) solution was added drop by drop and reaction mixture was stirred for 1 h. The pH of the mixture was adjusted to 9 by adding 60 mL of sodium hydroxide (0.5%) solution. Then the mixture was dried up and heated in air at 450°C for 20 min to oxidize Ce(OH)_3_ particles to CeO_2_ nanoparticles [12].

### Preconcentration method

The DSPME/EDXRF procedure is as follows: 1 mg of G/CeO_2_ was dispersed in 25 mL of analyzed sample solution. Sample pH was adjusted to optimized pH value using 0.1 mol∙L^−1^ of HNO_3_ and 0.1 mol∙L^−1^ of NH_3_aq solutions. After that the mixture was stirred for 5 min and then passed through a 0.45 μm membrane filter using a filtration assembly of 5 mm diameter. The filters with G/CeO_2_ and adsorbed analytes were dried and then analyzed by EDXRF.

## Results and discussion

### Characterization of G/CeO_2_ nanocomposite

**Fig. 1:**
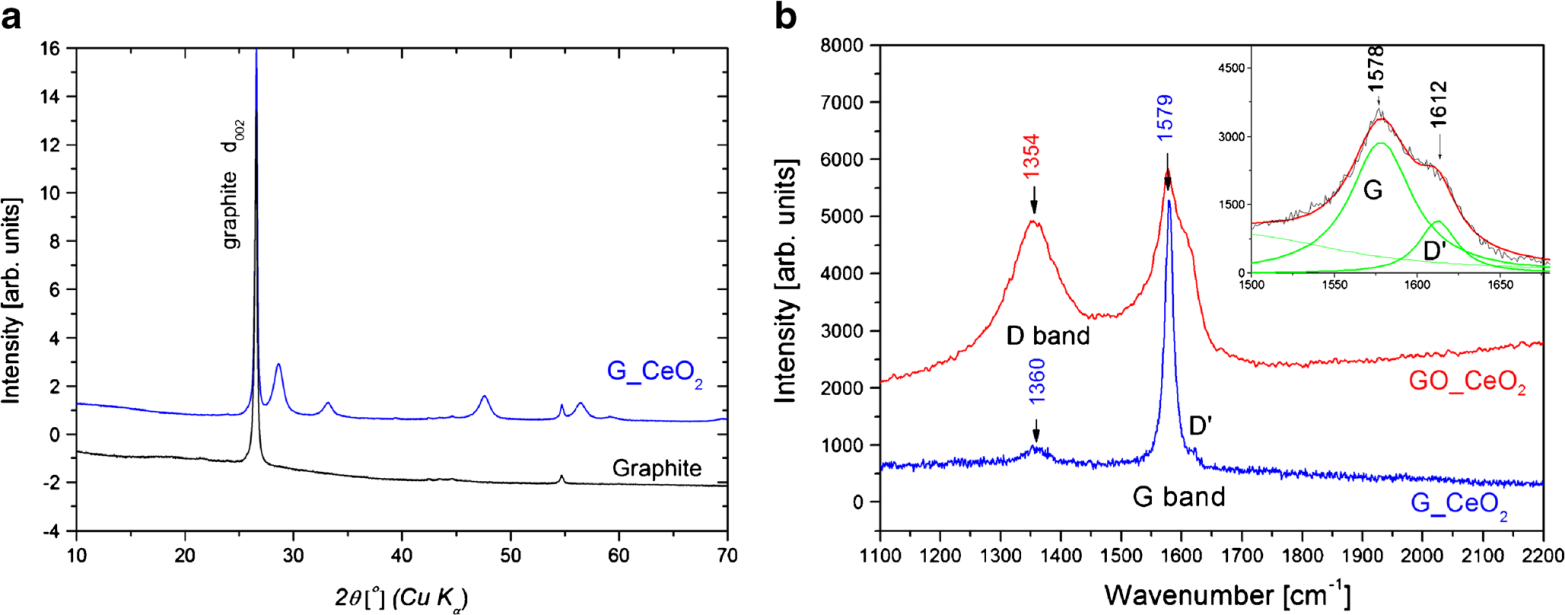
XRD patterns for graphite and G/CeO_2_ (a), and Raman spectra for G/CeO_2_ (b). Excitation with 830 nm

Before its use, the synthesised G/CeO_2_ nanocomposite was characterized by XRD and Raman spectroscopy. Figure 1a presents the XRD diffraction patterns of graphite and G/CeO_2_. The most pronounced is the diffraction peak of graphite at *2Θ* = 26.59^O^ that corresponds to coherently scattering hexagonal carbon layers with d_002_ spacing of 3.35 Å. The indexed diffraction patterns come from cubic *Fm*-3 *m* phase of CeO_2_ (cerianite, 28,709-ICSD) [13]. In G/CeO_2_ the mean size of the cerianite crystallites, calculated from the Scherer formula [14], is equal to 9 nm.

Figure 1b presents Raman spectrum for G/CeO_2_ powder. The main features of the spectra are two prominent bands which correspond to so-called G and D modes [15]. The G peak, which dominates in the spectrum of G/CeO_2_ at 1579 cm^−1^ is related to E_2g_ vibrational mode of ordered in-plane sp^2^ carbons and is characteristic for all sp^2^-hybridized carbon structures. The structural defects and impurities manifest as D and D’ peaks, linked to the breathing modes of carbon rings. In the infinite graphite and graphene layers the D peaks are not active. They appear with the reduction of the size of the crystallites and are always present in the nano-sized carbon structures [16]. The intensity and the widths of the G and D peaks carry information about the ‘amount’ of disorder in the sample. The broader the bands and the higher the I_D_/I_G_ intensity ratio the higher the disorder is [17]. In the Raman spectra of G/CeO_2_ the D and D’ peaks at 1360 and 1618 cm^−1^, respectively, are weak indicating the presence of a small number of the edge-carbon atoms in the sample.

**Fig. 2:**
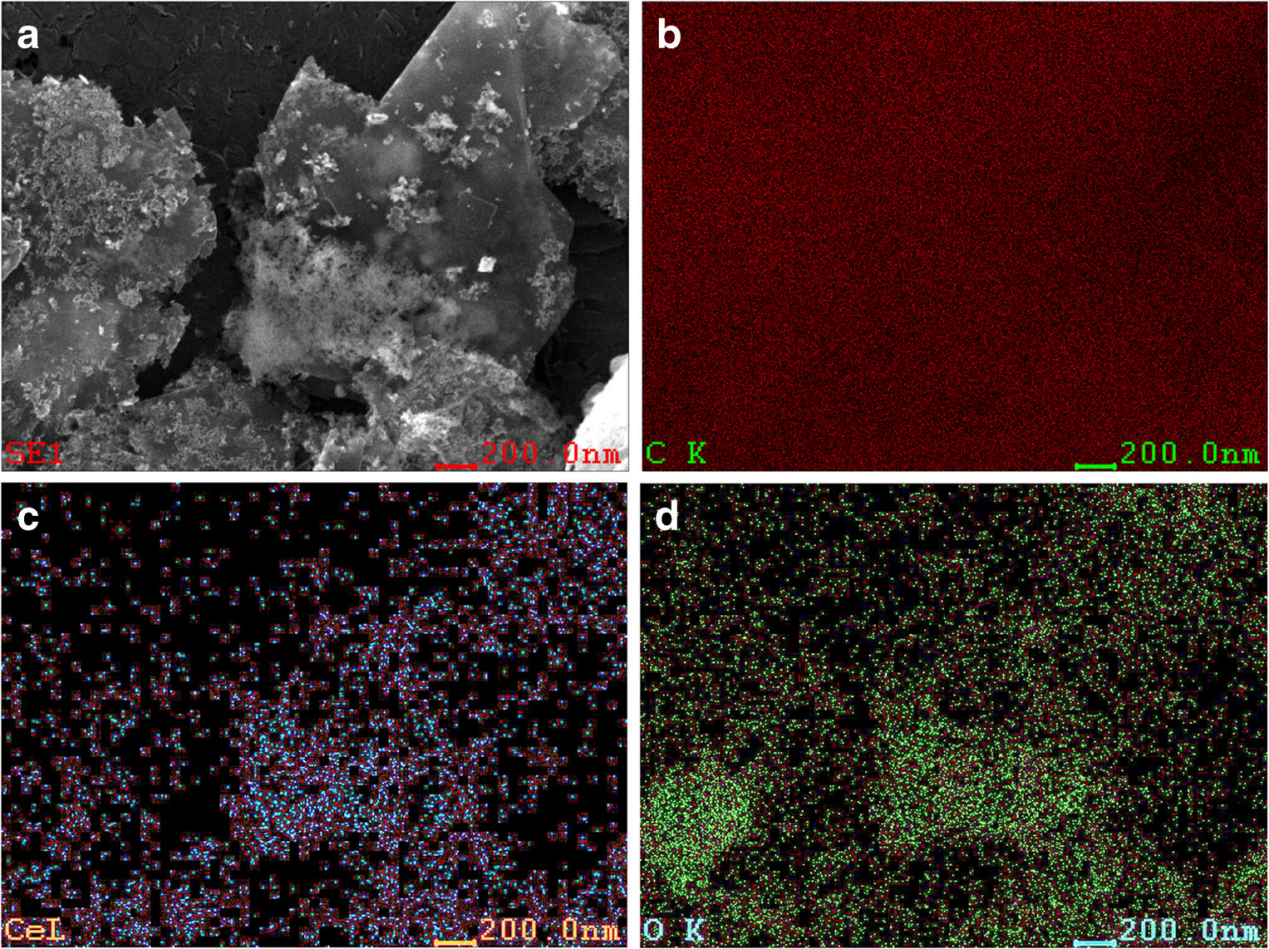
SEM images of synthesized G/CeO_2_ (**a**) and maps of the correlation between distribution of carbon (**b**), cerium (**c**) and oxygen (**d**) on the G/CeO_2_ surface

G/CeO_2_ was also observed by SEM. Figure 2a shows an SEM image of G nanosheets with nanoparticles of CeO_2_. The nanometric crystallites of CeO_2_ can be clearly seen on the surface of the graphene nanosheets. Figure 2b shows the distribution maps of C, Ce and O elements on the G/CeO_2_ surface. It can be seen the good correlation between distribution of cerium and oxygen on the surface of nanocomposite resulting from presence of CeO_2_ on graphene.

### Maximum adsorption capacity of the G/CeO_2_ nanocomposite

The maximum adsorption capacities of G/CeO_2_ toward As(V), Se(IV), Cu(II) and Pb(II) were 8.4, 14.1, 50.0 and 75.6 mg∙g^−1^, respectively, and they are considered successful values for a sorbent to be used in SPE of ultatrace metal ions. Respective data are given in the Electronic Supporting Material (ESM).

### Optimization of method

The following parameters were optimized: (a) pH; (b) effect of sample volume and contact time; (c) the effect of flow-rate; (d) effect of potentially interfering ions and organic matter. Respective data and Figures are given in ESM.

The following experimental conditions were found to give best results: (a) pH 4.0 for As(V), pH 3.0 for Se(IV), pH 6 for Cu(II) and Pb(II) sorption and determination; (b) best results for determination of As(V) and Se(IV) within 5 min can be obtained even if the sample volume is 500 mL. In case of the determination of Cu(II) and Pb(II) to obtain the best results within 5 min, the sample volumes should not exceed 250 mL and 100 mL, respectively; (c) The adsorption of Se(IV) reaches a maximum value of 100% using a flow rate of 0.3 mL∙min^−1^ - 4 mL∙min^−1^. The adsorption of As(V) is maximum at flow-rate of 0.3-0.7 mL∙min^−1^; (d) Na^+^ (<200 mg∙L^−1^), K^+^ (<200 mg∙L^−1^), Mg^2+^ (<200 mg∙L^−1^), Ca^2+^ (<200 mg∙L^−1^), NO_3_^−^ (<800 mg∙L^−1^), SO_4_^2−^ (<250 mg∙L^−1^), humic acid (<5 mg∙L^−1^), Al^3+^ (<2.5 mg∙L^−1^), Fe^3+^ (<2.5 mg∙L^−1^) and PO_4_^3−^ (<0.25∙mg L^−1^) do not influence the sorption of analytes. The influence of Al^3+^, Fe^3+^ and PO_4_^3−^ on analyte sorption are thoroughly discussed in ESM.

### Analytical performance

Analytical figures of merit of the procedure using G/CeO_2_ as solid adsorbent are presented in Table 1. The linearity was examined for the concentration of analytes in the range of 2–50.0 ng∙mL^−1^. The results show that the method is linear for the entire range of examined concentrations, which is confirmed by good correlation coefficients varying from 0.9982 (Cu) to 0.9997 (As, Se). The limits of detection were calculated from *LOD = (3/k)∙(B/t)*^*1/2*^, where k is the sensitivity of the method, B is the background count rate in counts s^−1^ and t is the counting time. The limits of detection in the range of 0.10 ng∙mL^−1^ - 0.21 ng∙mL^−1^ allow the application to the determination of As(V), Se(IV), Cu(II) and Pb(II) in water samples. The LODs are below the permissible levels in drinking waters (10, 50, 1300 and 15 ng∙mL^−1^ for As(III), Se(IV), Cu(II) and Pb(II), respectively [18]) according to The United States Environmental Protection Agency (US EPA). Such good LODs result from very low spectrum background arising from the use 100 μm Ag primary beam filter (EDXRF measurement) and from the possibility of using only 1 mg of the solid sorbent (DSPME) and in consequence obtaining thin samples of small diameter preferable for EDXRF analysis.

**Table 1:** Analytical figures of merit of the DSPME/EDXRF method Data for *n* = 10

Element	DSPME/EDXRF method				
	Linearity range, ng∙mL^−1^	Correlation coefficient, R	Sensitivity, mL∙ng^−1^∙s^−1^	LOD, ng∙mL^−1^	RSD, %
As(V)	2.0-50	0.9997	1.650	0.10	2.0
Se(IV)	2.0-50	0,9999	1.960	0.11	2.1
Cu(II)	2.0-50	0.9974	0.728	0.19	4.3
Pb(II)	2.0-50	0.9996	0.968	0.21	2.2

**Fig. 3:**
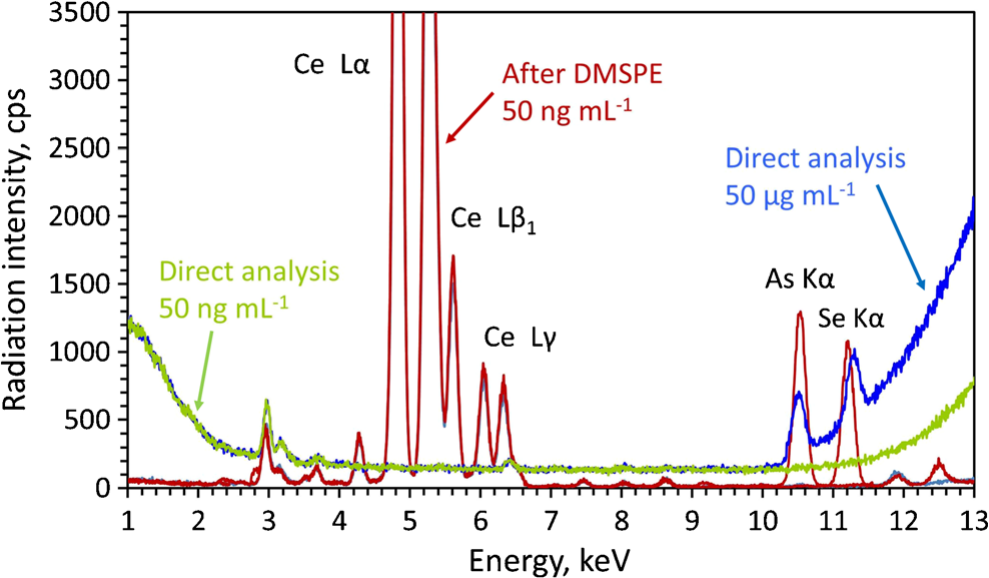
Comparison between EDXRF spectra obtained for the direct analysis of an aqueous standard solution containing 50 μg∙mL^−1^ (blue line) 50 ng∙mL^−1^ (green line) and after the DSPME procedure (red line)

The method is greatly simplified due to the elimination of the elution step. Therefore, whole precision of the method (including the sample preparation step) is very good and the RSD values are in the range of 2.0-4.3%. In Fig. 3 the benefits of using the method in terms of sensitivity are also displayed. As it is shown, the direct determination of 50 ng∙mL^−1^ As and Se in aqueous solution is practically impossible (the signals from As and Se are not present in the spectrum). Although the concentration of determined elements in a solution containing 50 μg∙L^−1^ is 1000 times higher, much worse signal-to-background ratio in comparison with the spectrum resulting from the analysis of a preconcentrated 50 ng∙mL^−1^ solution. These results highlight the high improvement of the sensitivity of the proposed method in comparison with the direct EDXRF analysis.

The accuracy of the method was verified by the analysis of the Certified Reference Material (CMR) of spring water (NIST 1640a). The results (see ESM) were in good agreement with the certified values for As (2.5%), Se (4%), Cu (2.5%) and Pb (1%) and recoveries for all determined elements were in range: 96% - 102%. The results show also that despite the rich matrix of the spring water As, Se, Cu and Pb ions were excellent adsorbed by the surface of G/CeO_2_, and quantitatively determined with DSPME/EDXRF method.

### Analysis of real samples

The method with G/CeO_2_ as a sorbent was used to analyze tap water, river water and seawater. Samples were spiked with analytes at the levels of 7.5 ng∙mL^−1^, 15 ng∙mL^−1^ and 30 ng∙mL^−1^. The results shown in Table 2 indicate that the recoveries (93% - 108%) are reasonable for determination of all analytes in tap water. In case of seawater analysis, a decrease in the recovery for Se(IV) is observed (~75%). Probably, the presence of sulphate ions in high concentration of 2.4 g∙L^−1^ (sulphate: selenite ratio (200,000:1)) effects negatively on the sorption of Se(IV). Taking into account the affinity of sulphate to selenate, interaction between both ions and nanocomposite may be similar. In case of river water the recovery of Se(IV) (>90%) is acceptable. The concentration of SO_4_^2−^ in river and lake water is much lower (reaches several tens of mg∙L^−1^ according to US EPA regulation [19]). As can be seen in Table 2 As(V), Cu(II) and Pb(II) can be also determined with good recoveries in river water (91%-104%).

**Table 2:** Determination of Se(IV), As(V), Cu(II) and Pb(II) in spiked water samples; *n* = 3; uncertainties correspond to one standard deviation

Sample	Added, ng mL^−1^	Determined, ng∙mL^−1^				Recovery, %			
		Se(IV)	As(V)	Cu(II)	Pb(II)	Se(IV)	As(V)	Cu(II)	Pb(II)
Seawater*	0	<LOD	<LOD	<LOD	<LOD	–	–	–	–
	7.5	5.9 ± 0.1	7.0 ± 0.2	6.9 ± 0.1	7.6 ± 0.4	78	93	92	102
	15	10.7 ± 0.2	14.3 ± 0.1	14.5 ± 0.1	14.4 ± 0.2	72	96	96	96
	30	22.0 ± 0.2	22.3 ± 0.2	22.3 ± 0.2	29.2 ± 0.2	73	75	74	97
River water	0	<LOD	<LOD	2.31 ± 0.01	0.93 ± 0.01	–	–	–	–
	7.5	6.9 ± 0.1	7.8 ± 0.1	9.8 ± 0.3	8.0 ± 0.1	92	104	99	95
	15	14.3 ± 0.2	13.95 ± 0.1	18.4 ± 0.1	15.9 ± 0.1	95	94	93	99
	30	28 ± 0.3	28.34 ± 0.3	33.3 ± 0.2	29.2 ± 0.3	93	93	94	94
Tap water	0	<LOD	<LOD	<LOD	<LOD	–	–	–	–
	7.5	7.1 ± 0.1	7.1 ± 0.2	6.9 ± 0.1	7.6 ± 0.2	95	94	93	105
	15	15.7 ± 0.2	15.8 ± 0.2	14.7 ± 0.1	14.7 ± 0.2	104	106	98	98
	30	29.7 ± 0.2	32.3 ± 0.3	32.0 ± 0.3	29.4 ± 0.3	99	108	107	98

*artificial seawater solution: 21.03 g NaCl, 3.52 g Na_2_SO_4_, 0.61 g KCl, 0.088 g KBr, 0.034 g Na_2_B_4_O_7_ ∙ 10H_2_O, 9.50 g MgCl_2_ ∙ 6H_2_O, 1.32 g CaCl_2_ ∙ 2H_2_O, 0.02 g SrCl_2_ ∙ 6H_2_O and 0.02 g NaHCO_3_ dissolved in 1 L of high purity water [20]

### Speciation analysis and method application

The possibility to use the method for inorganic selenium speciation was also tested. For that, the G/CeO_2_ nanocomposite was applied to the determination of Se(IV), Se(VI) and total Se at pH = 3. Recovery values were determined by spiking water samples with different Se(IV)/Se(VI) concentration ratios. Se(IV) was determined in one portion using this method, whereas total Se (the sum of Se(IV) and Se(VI)) was determined in another portion after reduction of Se(VI) to Se(IV) by gentle boiling in 5 M HCl medium for 15 min. The amount of Se(VI) was calculated by subtracting Se(IV) from the total amount of selenium. The concentration of Se(VI) was calculated as the difference. As it is shown in Table 3 Se(IV), Se(VI) and total Se can be successfully determined in water samples. A good agreement between the added and determined concentrations confirms the validity of the method for speciation analysis.

**Table 3:** Determination of Se(IV) and Se(VI) in spiked water samples; *n* = 3; the uncertainties correspond to one standard deviation

Added, ng∙mL^−1^		Found, ng∙mL^−1^		Recovery, %	
Se(IV)	Se(VI)	Se(IV)	Se(VI)	Se(IV)	Se(VI)
0	0	< DL	< DL	–	–
10.0	0	10.3 ± 0.1	< DL	103	–
0	10.0	< DL	10.3 ± 0.7	–	103
10.0	10.0	9.8 ± 0.2	9.8 ± 0.2	98	98

### Comparison of G/CeO_2_ with other sorbents based on carbon, as well as metal oxide nanomaterials

In the literature, the applications of G/CeO_2_ and modified G/CeO_2_ can be found mainly as sensors. The CeO_2_/G modified glassy carbon electrode (GCE) was used for thymol [21], nitrite [11], and cholesterol determination [22]. CeO_2_/reduced GO nanocomposites was used for the determination of fenitrothion [23], nitric oxide [24], and ssDNA [25]. Nafion coated CeO_2_/G was applied as amperometric biosensor for selective determination of dopamine [26]. The method uses G/CeO_2_ for the determination of metal ions. For comparison purposes, several experimental parameters, as well as analytical figures of merit together with those associated with other SPE methods using carbon, as well as oxide nanomaterials, in combination with other analytical techniques are summarized in Table 4.

**Table 4:** Summary of experimental details and analytical figures of merit of published SPE methods for preconcentration and determination of Cu(II), Pb(II), As(V) and Se(IV) using sorbents based on G, GO and/or metal oxide nanoparticles

Analyte	pH	Carbon sorbent	Mass of sorbent, mg	Contact time, min	Type of eluent	LOD, ng∙mL^−1^	RSD, %	Technique detection	Ref
As(V)	6.5	β-FeOOH@GO-COOH	1.0	15	NaOH/NaBH_4_ 2 mol L^−1^/2.0%	0.03	5.2	HG-AFS	[4]
As(V)	5.7	Alumina	10	15-1440	–	0.8	5	TXRF	[27]
Se(IV)	4.0	Magnetic-MWCNTs	10	20	NaOH 2.5 mol L^−1^	0.01	2.3	HG-AFS	[28]
Cu(II) Pb(II) La(III) Ce(III) Eu(III) Dy(III) Yb(III)	5.0	GO-TiO_2_	50	3.5	HNO_3_ 1 mol L^−1^	0.48 2.64 0.41 0.24 0.13 0.26 0.21	6.4 9.8 8.6 3.2 5.6 4.5 6.2	ICP-OES	[29]
Se(IV)	2.0	ZrO_2_/B_2_O_3_	200	50	HNO_3_ 1 mol L^−1^	0.12	4.0	ETAAS	[30]
Cr(III) Mn(II) Co(II) Ni(II) Cu(II) Cd(II) Pb(II)	10	Fe_3_O_4_@MOF^a^	10	11	HNO_3_ 0.5 mol L^−1^	0.6 0.9 1.0 0.9 0.3 0.4 0.7	2.9 5.5 3.9 6.4 6.2 5.6 3.9	ICP-OES	[6]
Fe(III) Co(II) Ni(II) Cu(II) Zn(II) Pb(II)	8	GO-EDA^b^	2	5	Solvent- free	0.07 0.10 0.07 0.08 0.06 0.10	4.1 4.8 4.4 4.5 5.0 3.6	EDXRF	[9]
Co(II) Ni(II) Cu(II) Zn(II) Pb(II)	5	GO	0.5	5	Solvent- free	0.5 0.7 1.5 1.8 1.4	4.3 4.5 2.5 5.1 3.4	EDXRF	[31]
Se(IV)	7.0	Modified nano-Al_2_O_3_	50	5	HNO_3_ 1 mol L^−1^	0.014	3.3	ICP-OES	[32]
Se(IV) Se(VI)	4.0	Nano-TiO_2_	100	15	NaOH 0.1 mol L^−1^	0.8 0.4		IC-CD	[33]
As(V)	7.3	TiO_2_	60	50	NaOH 0.5 mol L^−1^	40	19	ICP-OES	[34]
As(III) Se(IV) Sb(III)	8	ceria-coated silica–iron oxide	2.5	5 min ultrasonication	HNO_3_ 1 mol L^−1^	0.44 0.26 0.29	2.9 3.0 4.1	ICP-OES	[35]
						0.15 0.09 0.10		HG-ICP-OES	
Cu(II) Pb(II)	6.0	GO-TCC^c^	20	17	HNO_3_ 3 mol L^−1^	0.13 0.32	1.6 1.1	FAAS	[5]
Cu(II) Cd(II) Pb(II)	6.0	GO@Fe_3_O_4_@MBT^d^	15	4	HCl 0.4 mol L^−1^	0.24 0.19 0.35	3.2 3.5 2.4	FAAS	[7]
Cr(III) Cu(II) Zn(II) Cd(II) Pb(II)	5.8	mGO/SiO_2_@coPPy-Th^e^	22	6.5	HNO_3_ 0.82 mol L^−1^	0.36 0.15 0.23 0.21 0.65	6.0	FAAS	[10]
As(V) Se(IV) Cu(II) Pb(II)	4.0 3.0 6.0 6.0	G/CeO_2_	1.0	5	Solvent-free	0.10 0.11 0.19 0.21	2.0 2.1 4.3 2.2	EDXRF	This work

^a^Fe_3_O_4_@MOF - magnetic metal-organic frameworks

^b^GO-EDA - ethylenediamine-modified graphene oxide,^c^TCC - trithiocyanuric acid

^d^GO@Fe_3_O_4_@MBT - magnetic graphene oxide modified with 2-mercaptobenzothiazole, ^e^ mGO/SiO_2_@coPPy-Th-SiO_2_-coated magnetic graphene oxide modified with a pyrrole-thiophene

As can be seen in Table 4 one of the great advantage of the method is the small mass of G/CeO_2_ used as a sorbent (only 1 mg). Moreover the combination of DSPME with EDXRF analysis is especially profitable since the determination of trace amounts of metal ions is solvent-free. EDXRF allows analysis of solid sample and determining analytes on a G/CeO_2_ without elution. In general, the LODs are worse than the values obtained by ICP-MS and HG-AFS, but they are comparable or even better than other techniques such as ICP-OES, FAAS and TXRF. Moreover, the LODs are sufficient to determine ultratrace amounts of arsenic, selenium, copper and lead in the different type of waters according to the permissible levels of current legislation. Moreover, the sorption of analytes onto G/CeO_2_ DSPME is practically immediate. Thus, the sample preparation time is competitive in relation to other methods. A very important advantage of the method using G/CeO_2_ is the ability to determine both cationic and anionic forms of elements. The other methods presented in the Table 4 are directed either to the cationic forms of elements or only to anionic ones.

## Conclusion

A new method based on DSPME using G/CeO_2_ nanocomposite as a sorbent and EDXRF analysis has been developed for multielemental ultratrace determination of heavy metal ions and selenium speciation. G/CeO_2_ nanosheets appear as an attractive new nanomaterial in sorption of As(V), Se(IV), Cu(II), Pb(II) and Se(IV). G/CeO_2_ is characterized by selectivity towards Se(IV) in the presence of Se(VI). It is also worth noting here that the EDXRF allows direct analysis of solid samples, and therefore, the metal ions do not have to be eluted from the G/CeO_2_ before analysis. It is great advantage because it shortens the whole analysis time and reduces the cost. All of the mentioned qualities contribute to great suitability of DSPME with G/CeO_2_ in EDXRF measurement. The possibility of simultaneous determination in environmental waters of both anionic and cationic forms of metals distinguishes the method among others.

## Electronic supplementary material

ESM 1 (DOC 3782 kb)
