# Supplementary material for: Ceria nanoparticles deposited on graphene nanosheets for adsorption of copper(II) and lead(II) ions and of anionic species of arsenic and selenium
Source: Mikrochim Acta. 2018 Apr 23;185(5):264. doi: 10.1007/s00604-018-2806-6 (PMC5913377; doi:10.1007/s00604-018-2806-6)
Supplement: Supplementary file 1 — (DOC 3782 kb) [file 604_2018_2806_MOESM1_ESM.doc]

Electronic Supporting Material

Ceria nanoparticles deposited on graphene nanosheets for adsorption of copper(II) and lead(II) ions and of anionic species of arsenic and selenium

**Anna Baranik^a^, Anna Gagor^b^, Ignasi Queralt^c^, Eva Marguí^d^, Rafal Sitko^a^, Beata Zawisza^a*^**

^a^ University of Silesia, Institute of Chemistry, Szkolna 9, 40-006 Katowice, Poland

^b^ Institute of Low Temperature and Structure Research, Polish Academy of Sciences,

P.O. Box 1410, 50-950 Wrocław, Poland

^c^ Institute of Environmental Assessment and Water Research, Dep. of Geosciences, IDAEA-CSIC, Jordi Girona St., 18-26, 08034 Barcelona, Spain

^d^ Department of Chemistry, University of Girona, Faculty of Sciences, C/M.Aurèlia Campmany, 69, Girona, Spain

* Corresponding author. Tel.: +48 32 359 2258; fax: +48 32 2599978; E-mail address: beata.zawisza@us.edu.pl

**Experimental**

**Batch adsorption experiment**

The maximum adsorption capacity for G/CeO­_2_ toward As(V), Se(IV), Cu(II) and Pb(II) was carried out at pH 4.0, 3.0, 6.0 and 6.0, respectively (see pH study section). The experimental procedure consists of introducing 1 mg of G/CeO_2_ to 25mL of aqueous solution and the subsequent dispersed in an ultrasonication bath for 15 min. Then the ions of arsenic, selenium, copper and lead were introduced into the suspentions of G/CeO­_2_. The initial concentration of ions were: 0.05-6.97 μg mL^-1^ of As(V), 0.22-5.50 μg mL^-1^ of Se(IV), 0.65-6.65 μg mL^-1^ of Cu(II) and 0.05-3.89 μg mL^-1^ of Pb(II). The pH values of the suspensions were adjusted by solutions of HNO_3_ and NH_3_aq. The samples were stirring for 1.5 h to reach the adsorption equilibrium. At the end suspensions were filtered thought the 0.45 μm membrane filters and the obtained filtrates were analysed by ICP-OES technique.

The amount of metal ions adsorbed on G/CeO_2_ (mg g^-1^) were calculated from the difference between the initial concentration C_0_ (mg L^-1^) in aqueous solution and the equilibrium concentration C_e_ (mg L^-1^) determined in the filtrate. The relationship between C_0_ and C_e_ is expressed as follows: *q_e_ = (C_0_ – C_e_)V/m_adsorbent_*, where *V* is the volume of the suspension, and *m_adsorbent_* is the mass of G/CeO_2_. The recovery is given by: *Recovery (%) = 100(C_0_ – C_e_)/C_0_.*

**Speciation analysis**

Total selenium (sum of Se(IV) and Se(VI)) was determined after pre-reduction of Se(VI) to Se(IV) by gentle boiling in 5 M HCl medium for 15 min [1]. The amount of Se(VI) was calculated by subtracting Se(IV) from the total amount of selenium.

**Results and Discussion**

**pH study**

The most important factor with respect to the adsorption of metal ions is the acidity of the sample solution. pH studies in the range of 1-9 showed that As(V), Se(IV), Cu(II) and Pb(II) ions were adsorbed with the highest recoveries (92-100%) at pH 4.0, 3.0, 6.0 and 6.0, respectively (see **Fig.S1**). Moreover, as it can be seen in the **Fig. S1** the speciation analysis of selenium on G/CeO_2_ is also possible. At pH 3.0-4.0 the recovery for Se(IV) was above 90% while for Se(VI) it was below 10%. In case of arsenic both species are adsorbed with different recoveries, but the difference in recovery between As(III) and As(V) is not sufficient to carry out the speciation analysis. Thus, total arsenic is recommended to be determined after pre-oxidation of As(III) to As(V). For this purpose 150 μL of 10^-4^ mol L^-1^ of KMnO_4_ [2] was introduced to the sample before DSPME/EDXRF.

**
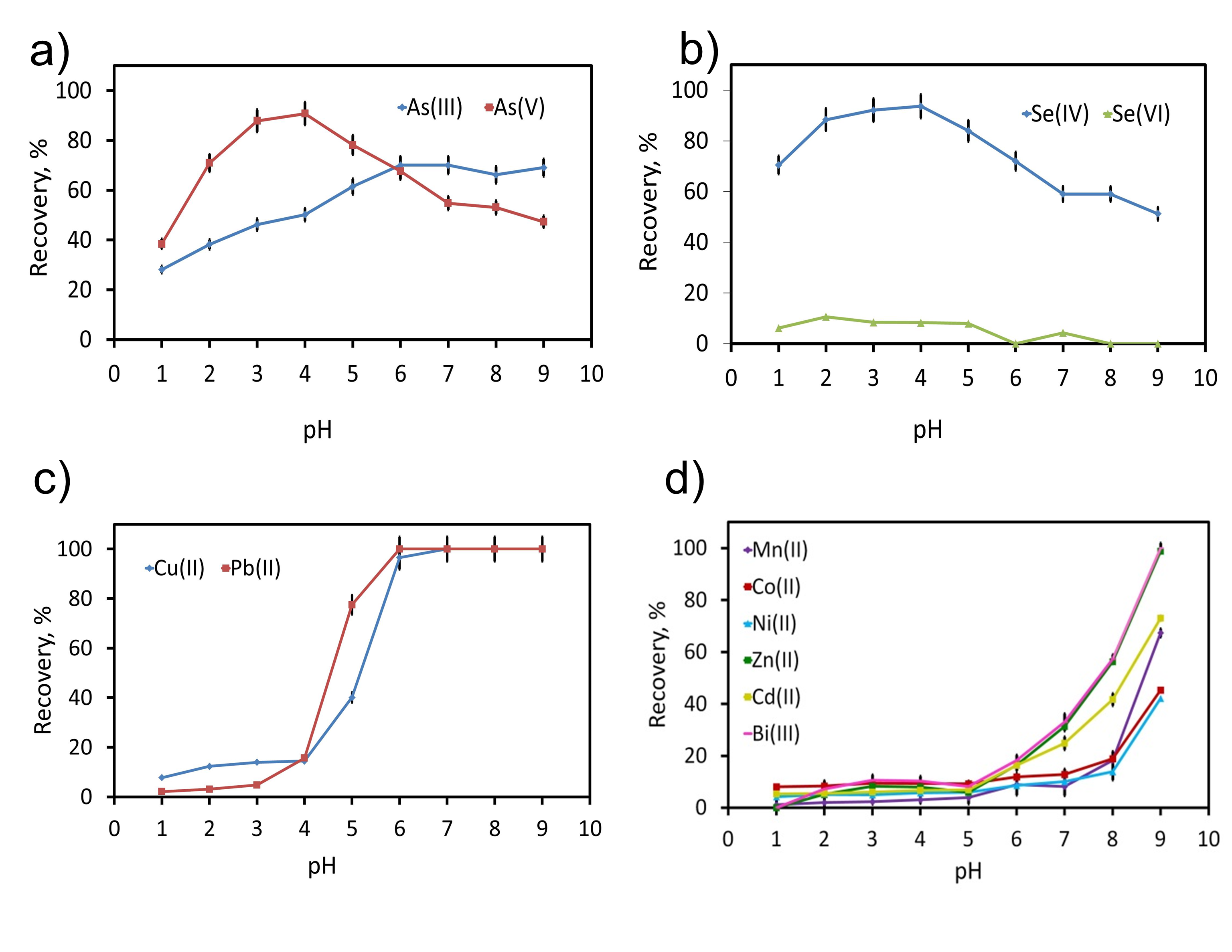
**

**Fig. S1** Effect of sample pH on the recovery of As (a), Se (b), Cu and Pb (c), Mn, Co, Ni, Zn, Cd, Bi (d). (Experimental conditions: mass of G/CeO_2_ = 1 mg; the sample volume = 25mL; the stirring time = 90 min, the metal concentration = 250 ng mL^-1^, n=6.)

The adsorption of anions by G/CeO_2_ is based on the reaction between anions and the –CeOH or –CeOH_2_^+^ on the adsorbent surface [3]. These groups are responsible for both electrostatic interaction between H_2_AsO_4_^-^, H_2_SeO_3_ and HSeO_3_^-^. As(V) and Se(IV) ions were adsorbed with the highest recoveries (96-103%) at pH 4.0 and 3.0, respectively. Moreover, the speciation analysis of selenium on G/CeO_2_ is also possible. At pH 3.0-4.0 the recovery for Se(IV) was above 90% while for Se(VI) it was below 10%. In case of arsenic both species are adsorbed with different recoveries, but the difference in recovery between As(III) and As(V) is not sufficient to carry out the speciation analysis. Thus, total arsenic is recommended to be determined after pre-oxidation of As(III) to As(V). For this purpose 150 μL of 10^-4^ mol L^-1^ of KMnO_4_ was introduced to the sample before DSPME/EDXRF. Although, in the acid solution the G/CeO_2_ achieved positive charges Cu^2+^ and Pb^2+^ were adsorbed. The predominant species of copper and lead are positively charged Pb^2+^, Pb(OH)^+^ as well as Cu^2+^, Cu(OH)^+^ at pH < 6. Therefore, the metal ions can be chelated by the neighboring carboxyl and hydroxyl groups remaining on the nanocomposie surface. The best adsorption of Pb(II) and Cu(II) ions is observed at pH ≥ 6. Then the surface charge of nanocomposie is negative. Thus, the electrostatic interaction between nanocomposite and Pb^2+^ as well as Cu^2+^ can be also observed. In basic solution the metal ions begin to form a precipitate depending on initial metal concentration. Finally, taking into consideration both the high adsorption of metal ions and the prevention of the precipitation of metal hydroxides, the subsequent experiments and analysis of real samples were performed at pH 6. The carried out studies also indicate, that Mn(II) is quantitatively preconcentrated on the adsorbent at pH 9. Under these conditions, the manganese becomes to precipitate as hydroxide. The recoveries of Co(II), Ni(II), Zn(II), Cd(II) and Bi(III) are not satisfied in the whole studied pH range. Therefore, they were not considered in further studies.

**Maximum adsorption capacity of the G/CeO_2_ nanocomposite**

Analytes adsorption on the synthesized nanocomposite was studied adjusting the experimental data to Langmuir [4,5] and Freundlich [6] isotherm models. Experimental parameters for both models are displayed in **Table S1**. A better correlation coefficient was obtained for the Langmuir model suggesting a chemical adsorption process. The maximum adsorption capacities (q_max_) of G/CeO_2_ nanocomposite toward As(V), Se(IV), Cu(II) and Pb(II) were calculated by Langmuir model at pH 4.0, 3.0, 6.0 and 6.0 for As(V), Se(IV), Cu(II) and Pb(II), respectively.

**Table S1** Parameters for Langmuir and Freundlich models

| Analyte | Langmuir model | | | Freundlich model | | |
| --- | --- | --- | --- | --- | --- | --- |
|  | q_max_, mg g^-1^ | K_L_, L g^-1^ | R | n | K_F_, mg^1-n^ L^n^ g^-1^ | R |
| As(V) | 8.4 | 2.5 | 0.9676 | 5.6 | 5.9 | 0.9709 |
| Se(IV) | 14.1 | 1.8 | 0.9560 | 4.4 | 9.1 | 0.9462 |
| Cu(II) | 50.0 | 0.8 | 0.9890 | 2.5 | 21.3 | 0.9694 |
| Pb(II) | 75.6 | 6.6 | 0.9092 | 10.2 | 64.8 | 0.8868 |

**Optimization of method.**

**Effect of sample volume and contact time**

The sample volume as well as the contact time between a sorbent and analyte solution have a significant impact on adsorption percentage of analytes. The effect of sample volume and contact time for the determination of As(V), Se(IV), Cu(II) and Pb(II) were investigated in the range of 10-500 mL and 5-120 min at pH 4.0, 3.0, 6.0 and 6.0, respectively. As can been seen in **Fig. S2**, As(V) and Se(IV) were adsorbed by G/CeO_2_ with recoveries above 95% and 90%, respectively in the whole range of sample volume and contact time considered. Cu(II) ions were adsorbed with recoveries above 98% when using sample volumes in the range of 10-250 mL independently of the stirring time. For higher sample volumes the recovery of Cu(II) decreases. For instance, a recovery of 75% was obtained using a sample volume of 500 mL and a stirring time of 120 min. A similar trend was observed for Pb(II) ions determination. Pb(II) ions were adsorbed with recoveries near 95% from solution volumes in the range of 10-100 mL in the whole range of time but to achieve recovery values above 90% when using 250 mL and 500 mL the stirring time should be longer than 5 min and 30 min, respectively.

**
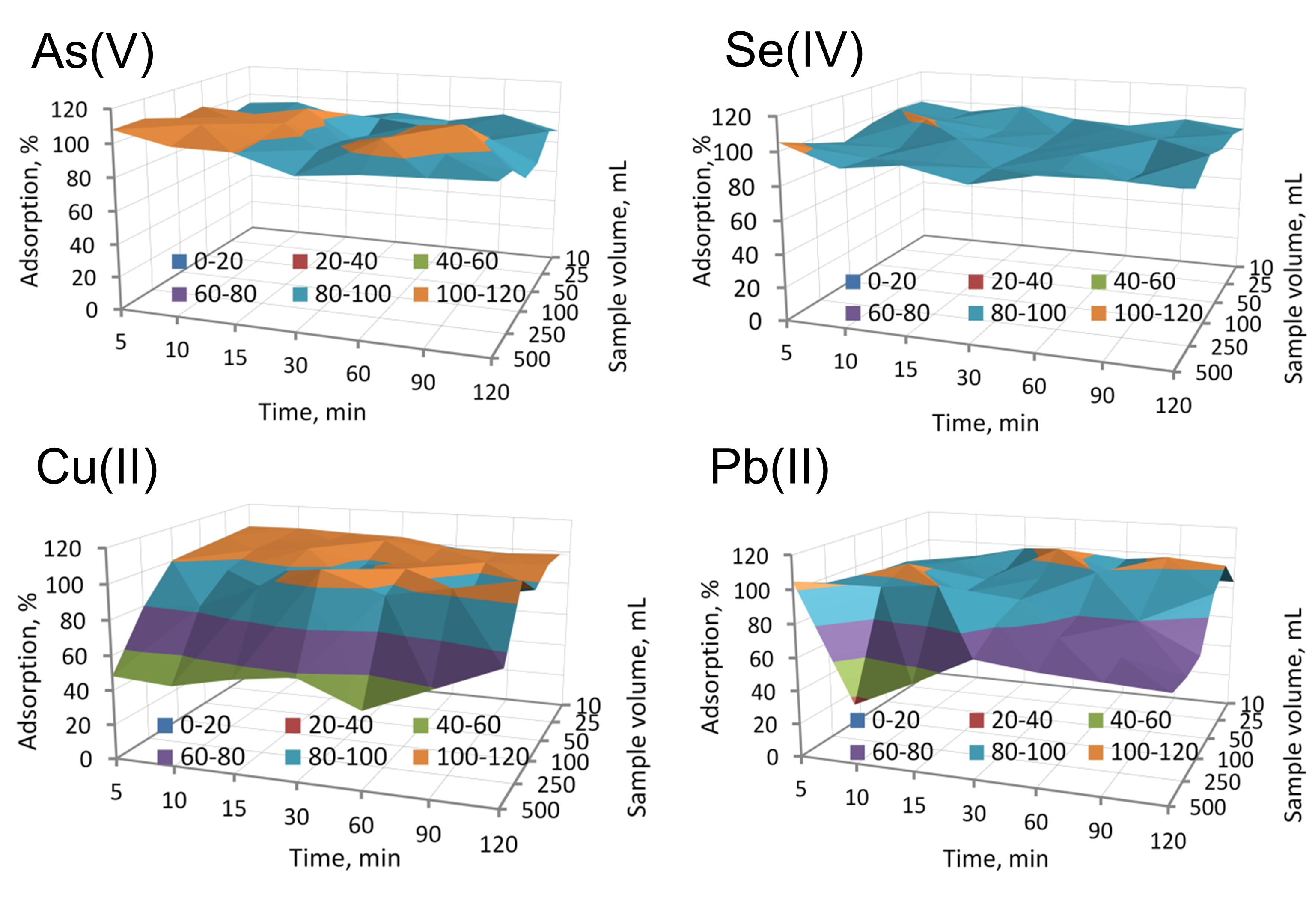
**

**Fig. S2** Effects of sample volume and contact time on the recovery of arsenic (a), selenium (b), copper (c), lead (d). (Experimental conditions: mass of G/CeO_2_ = 1 mg; c_analyte_ = 10 ng mL^-1^, pH = 4 (As), 3 (Se), 6 (Cu, Pb), n=3)

The excellent adsorptive properties of G/CeO_2_ nanocomposite motivated us to prepare a new membranes loaded with this adsorbent and their application under flow conditions. In this case, the G/CeO_2_ nanocomposite was deposited on membranes via vacuum filtration, and next the analyzed solution was passed through these new membranes. As an example, the effect of flow-rate for Se(IV) and As(V) ions is presented in **Fig. S3**. As it can be seen, the adsorption of Se(IV) ions reaches a maximum value of 100% using a flow rate of 0.3 mL min^-1^ and remains constants up to 4 mL min^-1^. The adsorption of As(V) ions reaches also maximum value of 100% at flow-rate of 0.3-0.7 mL min^-1^ but it decreases using flow rates higher than 1.3 mL min^-1^. It indicates that the adsorption of As(V) under flow conditions needs at least 5 times longer than DSPME to achieve the same high recovery of 100 %.


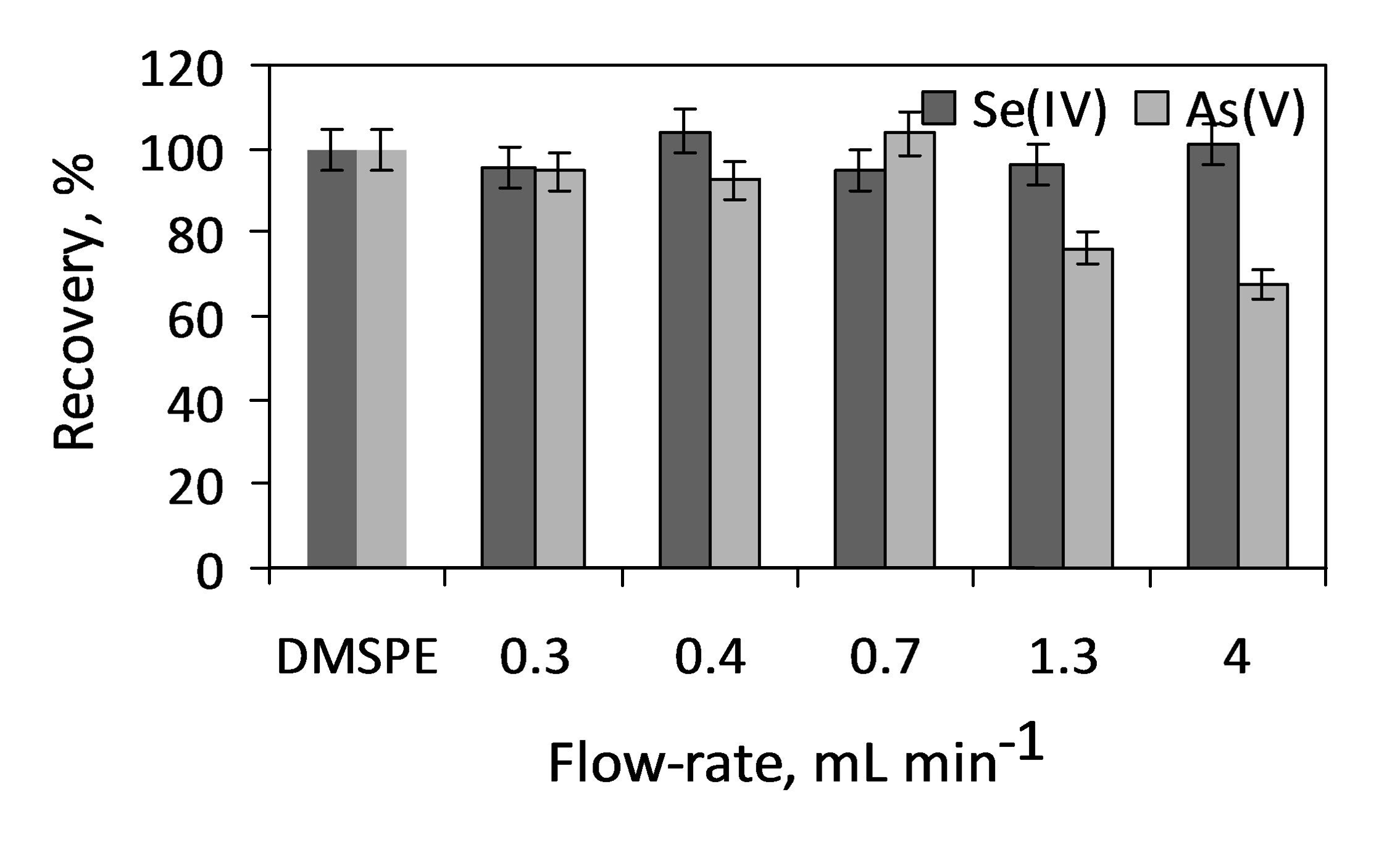


**Fig. S3** The effect of flow-rate for Se(IV) and As(V) ions adsorption on G/GeO_2_ nanocomposite deposited on a membrane (Experimental details: G/CeO_2_ membrane of mass per unit area of 0.32 mg cm^2^, c_analyte_ = 40 ng mL^-1^, pH=3.0 and 4.0 for Se(IV) and As(V), respectively, V=25 mL).

**Effect of potentially interfering ions and organic matter**

As shown in **Table S2**, the addition of Na^+^, K^+^, Mg^2+^, Ca^2+^, NO_3_^-^ and SO_4_^2-^ ions as well as humic acid does not influence the sorption of As(V), Se(IV), Cu(II) and Pb(II) on the G/CeO_2_ in the studied concentration ranges. The recoveries of all determined elements were in the range 91-106%. The addition of 10 μg mL^-1^ of Al^3+^ ions does not affect the high recovery of As(V), Se(IV) and Pb(II) but influences the recovery of Cu(II) (74%). Unfortunately, the presence of Fe^3+^ at a concentration level of 10 μg mL^-1^ effects negatively the sorption of As(V) and Se(IV) and the obtained recoveries were decreased up to a value of 31% in the case of As(V). Finally, the presence of PO_4_^3-^ ions in a concentration ratio PO_4_^3-^ : analyte (25:1) effect negatively only on the sorption of As(V). This fact can be explained taking into account the affinity of AsO_4_^3-^ to PO_4_^3-^ and similar interaction between both ions and nanocomposite.

**Table S2** Influence of interfering ions on the recovery of As(V), Se(IV), Cu(II) and Pb(II) on G/CeO_2_. (Experimental conditions: 1 mg of G/CeO_2_, 10 ng mL^-1^ of analytes, 25 mL of sample volume, 5 min of stirring time, n=6)

| Interferent | Concentration  of interferent,  μg mL^-1^ | Ratio of analyte and interferent concentration | Recovery, % ± RSD | | | |
| --- | --- | --- | --- | --- | --- | --- |
|  |  |  | As(V) | Se(IV) | Cu(II) | Pb(II) |
| Na^+^ | 200 | 1 : 20 000 | 102 ± 0.2 | 105 ± 0.5 | 101 ± 0.2 | 101 ± 0.1 |
| K^+^ | 200 | 1 : 20 000 | 106 ± 0.2 | 96 ± 0.1 | 95 ± 0.5 | 97 ± 0.2 |
| Mg^2+^ | 200 | 1 : 20 000 | 101 ± 0.3 | 100 ± 0.2 | 92 ± 0.2 | 103 ± 0.4 |
| Ca^2+^ | 200 | 1 : 20 000 | 106 ± 0.3 | 94 ± 0.5 | 95 ± 0.1 | 101 ± 0.2 |
| Al^3+^ | 2.5 | 1 : 250 | 103 ± 0.4 | 102 ± 0.5 | 94 ± 0.8 | 97 ± 0.3 |
| Al^3+^ | 10 | 1 : 1000 | 97 ± 0.5 | 104 ± 0.7 | 74 ± 0.5 | 99 ± 0.2 |
| Fe^3+^ | 2.5 | 1 : 250 | 92 ± 0.7 | 91 ± 0.8 | 94 ± 0.2 | 92 ± 0.2 |
| Fe^3+^ | 5 | 1 : 500 | 73 ± 0.1 | 73 ± 0.7 | 93 ± 0.8 | 100±0.5 |
| Fe^3+^ | 10 | 1 : 1000 | 31 ± 0.2 | 72 ± 0.1 | 93 ± 0.7 | 89 ± 0.8 |
| SO_4_^2-^ | 250 | 1 : 25 000 | 95 ± 0.4 | 91 ± 0.1 | 101 ± 0.7 | 98 ± 0.3 |
| PO_4_^3-^ | 0.25 | 1 : 25 | 95 ± 0.5 | 102 ± 0.4 | 97 ± 0.3 | 99 ± 0.9 |
| PO_4_^3-^ | 1.0 | 1 : 100 | 47 ± 0.2 | 98 ± 0.5 | 96 ± 0.9 | 102 ± 0.4 |
| NO_3_^-^ | 800 | 1 : 80 000 | 105 ± 0.1 | 101 ± 0.2 | 102 ± 0.3 | 100 ± 0.2 |
| HA | 5.0 | 1 : 500 | 103 ± 0.7 | 100 ± 0.9 | 102 ± 0.5 | 96 ± 0.2 |

**Analysis of CRM**

The accuracy of the method was verified by the analysis of the Certiﬁed Reference Material (CMR) of spring water (NIST 1640a). The results are given in **Table S3**.

**Table S3** Analysis of the Certiﬁed Reference Material Spring water (NIST 1640a), n=3

| **Spring water (NIST 1640a)** | | | | | |
| --- | --- | --- | --- | --- | --- |
| Major element of matrix, mg L^-1^ | Tace element of matrix,  μg L^-1^ | Analyte | Certified concentration, μg L^-1^ | Determined concentration, μg L^-1^ | Recovery,  % |
| Ca (5.615±0.021), Mg (1.0586±0.0041), K (0.5799±0.0023), Si (5.210±0.021), Na (3.137±0.031) | Al (53.0±1.8),  Ba (151.80±0.83),  B (303.1±3.1),  Cr (40.54±0.30),  Co (20.24±0.24),  Fe (36.8±1.8),  Mn (40.39±0.36),  Mo (45.60±0.61),  Ni (25.32±0.14),  Sr (126.03±0.27),  U (25.35±0.27),  V (15.05±0.25),  Zn (55.64±0.35) | As Se Cu Pb | 8.075±0.070  20.13±0.17  85.75±0.51  12.101±0.050 | 8.3±0.14  19.3±0.4  87.8±0.2  12.0±0.5 | 102  96  102  99 |

**References**

1. Xia L, Hu B, Jiang Z, Wu Y, Chen R, Li L (2006) Hollow fiber liquid phase microextraction combined with electrothermal vaporization ICP-MS for the speciation of inorganic selenium in natural waters. J Anal Atom Spectrom 21:362–365

2. Jitmanee K, Oshima M, Motomizu S (2005) Speciation of arsenic(III) and arsenic(V) by inductively coupled plasmaatomic emission spectrometry coupled with preconcentration system. Talanta 66:529–533

3. Peng X, Luan Z, Ding J, Di Z, Li Y, Tian B (2005) Ceria nanoparticles supported on carbon nanotubes for the removal of arsenate from water. Mater Lett 59:399–403

4. Langmuir I (1916) The constitution and fundamental properties of solids and liquids. J Am Chem Soc 38:2221–2295

5. Langmuir I (1918) The adsorption of gases on plane surfaces of glass, mica and platinium. J Am Chem Soc 40:1361–1403

6. Freundlich HMF (1916) Uber die adsorption in lasugen. Z Phys Chem 57:385–470
